# Transcriptomics‐guided identification of an algicidal protease of the marine bacterium *Kordia algicida* OT‐1

**DOI:** 10.1002/mbo3.1387

**Published:** 2023-10-10

**Authors:** Kristy S. Syhapanha, David A. Russo, Yun Deng, Nils Meyer, Remington X. Poulin, Georg Pohnert

**Affiliations:** ^1^ Institute for Inorganic and Analytical Chemistry, Bioorganic Analytics Friedrich Schiller University Jena Jena Germany; ^2^ Department of Chemistry and Biochemistry, Center for Marine Science University of North Carolina Wilmington Wilmington North Carolina USA

**Keywords:** algicidal bacteria, algicidal protease, diatoms, microbial interactions, phytoplankton, transcriptomics

## Abstract

In recent years, interest in algicidal bacteria has risen due to their ecological importance and their potential as biotic regulators of harmful algal blooms. Algicidal bacteria shape the plankton communities of the oceans by inhibiting or lysing microalgae and by consuming the released nutrients. *Kordia algicida* strain OT‐1 is a model marine algicidal bacterium that was isolated from a bloom of the diatom *Skeletonema costatum*. Previous work has suggested that algicidal activity is mediated by secreted proteases. Here, we utilize a transcriptomics‐guided approach to identify the serine protease gene *KAOT1_RS09515*, hereby named *alpA1* as a key element in the algicidal activity of *K. algicida*. The protease AlpA1 was expressed and purified from a heterologous host and used in in vitro bioassays to validate its activity. We also show that *K. algicida* is the only algicidal species within a group of four members of the *Kordia* genus. The identification of this algicidal protease opens the possibility of real‐time monitoring of the ecological impact of algicidal bacteria in natural phytoplankton blooms.

## Graphical abstract

Algicidal bacteria shape the plankton communities of the oceans by lysing microalgae and consuming the released nutrients. *Kordia algicida* is an environmentally relevant marine bacterium whose algicidal activity is mediated by secreted proteases. In this study, we utilize a transcriptomics‐guided approach to identify the secreted serine protease AlpA1 as a key factor in the algicidal process. This discovery offers new approaches for the real‐time monitoring and manipulation of algicidal bacteria in algal blooms.

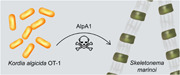

## 1 INTRODUCTION

Algicidal bacteria have attracted interest for their potential to act as biotic regulators of harmful algal blooms (HABs) and as potential tools in biotechnological applications (Doucette et al., 1999; Mayali & Azam, 2004; Meyer et al., 2017). They are commonly found associated with late‐stage phytoplankton blooms (Imai et al., 2001; Kim et al., 1998; Skerratt et al., 2002); however, their ecological role is still yet to be clearly defined (Coyne et al., 2022; Meyer et al., 2017; Wang et al., 2020). It has been suggested that algicidal bacteria play a dominant role in the microbial loop and influence the global cycling of organic carbon in the aquatic environment (Azam et al., 1983). Additionally, algicidal bacteria may also influence the succession of natural phytoplankton communities (Bigalke et al., 2019; Onishi et al., 2021).

Algicidal bacteria act by either direct contact with algal cells or by the release of diffusible algicidal compounds (Mayali & Azam, 2004; Meyer et al., 2017). The majority of knowledge about released algicidal compounds derives from work done with cell‐free filtrates (Coyne et al., 2022; Meyer et al., 2017; Wang et al., 2020). However, due to their diverse nature, there are no standardized methods to elucidate algicidal compounds, which can range from small molecules (Sakata et al., 2011; Ternon et al., 2018; Wu et al., 2011), to peptides (Banin et al., 2001; Hibayashi & Imamura, 2003; Imamura et al., 2000) to enzymes (Kohno et al., 2007; Lee et al., 2000, 2002; Paul & Pohnert, 2011).

Algicidal extracellular proteins may seem energetically costly for the producing microorganism, but they might fulfill a dual function in lysing or inhibiting the algae and in the breakdown of cellular content to release essential nutrients. Identification of active enzymes can be supported by molecular techniques that also provide insights into their genetic regulation. In recent years, transcriptomics has been used to broadly identify enzymes upregulated during algicidal activity (Zhang et al., 2022, 2023). Multiple regulated metabolic pathways were revealed in the transcriptomic analysis of the algicidal mechanism of the bacterium *Brevibacillus laterosporus* strain Bl‐zj against the cyanobacterium *Microcystis aeruginosa* strain FACHB 095 (Zhang et al., 2022). The co‐cultured bacteria differentially expressed genes involved in amino acid, carbohydrate, and lipid metabolism, with significantly increased expression of genes involved in valine, leucine, isoleucine, and fatty acid degradation. These could deliver the energy required to produce algicides. The upregulation of secreted hydrolytic enzymes, antibiotics, proteases, and other secondary metabolites was hypothesized to aid in the destruction of algal cells (Zhang et al., 2022). An integrated transcriptomic and metabolomic study was used to characterize the algicidal process of the bacterium *Enterobacter hormaechei* strain F2 in co‐cultivations with *M. aeruginosa* strain FACHB‐315 (Zhang et al., 2023). Analysis of differentially expressed genes during the algicidal process revealed enrichment of energy metabolism and aromatic amino acid metabolism‐related pathways. Integration with metabolomic analysis revealed significant changes in peptides, co‐enzymes, vitamins, and energy substances, and revealed potential algicides. An enrichment of chemotaxis‐related genes alluded to the direct algicidal mechanism used by this bacterium (Zhang et al., 2023).

Exploring the genome of *B. laterosporus* Bl‐zj revealed 18 potential algicidal proteases (Zhang et al., 2021). Bioassays have led to the discovery of an increase in the activity of enzymes such as β‐glucosidase (Kim et al., 2009), chitinases (Li et al., 2016), and l‐amino acid oxidases (Chen et al., 2010, 2011) during the algicidal process. Other studies predicted enzymatic algicidal activity based on the evaluation of extracellular degradative enzymes (Mayali et al., 2008; Zhou et al., 2021). Few studies have identified algicidal proteases at the genomic level in algicidal bacteria, with corresponding in vitro confirmation (Kohno et al., 2007).

In our study, we focus on the algicidal marine bacterium *K. algicida* OT‐1. *K. algicida* was first reported by Sohn et al. (2004) as a gram‐negative marine bacterium of the *Flavobacteriaceae* family isolated from a bloom of the diatom *Skeletonema costatum* (Sohn et al., 2004). In a natural community enclosure experiment, *K. algicida* shifted the plankton population and accelerated plankton succession by the removal of a dominant, susceptible alga (Bigalke et al., 2019). The *K. algicida* genome was reported in 2011 and interestingly revealed gliding motility genes, although the bacterium is nonmotile and nongliding (Lee et al., 2011). The bacterium has a broad target range, with activity reported against diatoms, dinoflagellates, and raphidophytes (Sohn et al., 2004). Paul and Pohnert (2011) initially hypothesized that the algicidal compound released by *K. algicida* is a serine protease that may be regulated by a quorum‐sensing mechanism. Quorum sensing is a density‐dependent bacterial process of cell–cell communication which is based on the release of signaling molecules that build up in their concentration in denser cultures (Dow, 2021). Once a critical concentration is reached, gene regulation, and as a result metabolic responses, of the bacteria is triggered. The diatom *Chaetoceros didymus* is resistant to *K. algicida*, producing algal proteases as part of a defensive mechanism against *K. algicida* (Paul & Pohnert, 2013).

Here, we present the identification of a gene coding for an algicidal protease in *K. algicida*. The discovery of active, inactive, and inducible modes of algicidal activity in this bacterium facilitated the use of transcriptomics to identify algicidal candidates. Further analysis narrowed the candidates to a single protease, AlpA1, that was obtained in its active state by expression of the gene in *Escherichia coli*. Finally, we showed that other members of the *Kordia* genus do not have algicidal activity despite the presence of AlpA1 homologs in their genomes.

## 2 METHODS

### 2.1 Strains and growth conditions

*K. algicida* OT‐1 (accession number NBRC 1000336), *Kordia aestuarivivens* (accession number NBRC 114499), *Kordia periserrulae* (accession number NBRC 106077), and *Kordia* sp. (accession number NBRC 113026) were obtained from the Biological Resource Center, NITE (NBRC), and stored as a cryo archive. Bacterial cultures for each experiment were initiated from cryo archives by streaking onto marine broth agar plates and incubating at 28°C for 2−3 days. Single colonies were then transferred to marine broth (MB) (Carl Roth) and grown at 28°C with 80−100 rpm shaking until the mid to late exponential phase was reached. *Skeletonema marinoi* (accession number RCC75) was purchased from the Roscoff Culture Collection and maintained in artificial seawater media (ASW), according to Maier and Calenberg (1994), with a salinity of 35 PSU, at 13°C, 14:10 light‐dark cycle with light intensity range 15−30 µmol/m^2^/s. The same recipe for ASW was used for *Kordia* experiments.

### 2.2 *K. algicida* growth analysis

Growth of *K. algicida* was monitored via periodic optical density measurements at 550 nm on a Genesys 10S ultraviolet–Vis spectrophotometer (Thermo Fisher Scientific) in tandem with algicidal activity bioassays, as described below. Four biological replicates were inoculated with single colonies in 20 mL MB. Growth was monitored starting at 12 h postinoculation, and subsequently, every 3 h until 48 h. A final measurement was taken at 62 h. The algicidal activity, as described below, was assessed at each timepoint. Following inactivation, subsequent dilution in MB did not restore algicidal activity. Having hypothesized that algicidal activity requires nutrient starvation, *K. algicida* was incubated in MB:ASW (1:10 v/v). Consequently, algicidal activity was recovered.

### 2.3 Algicidal activity bioassay

The algicidal activity was determined by calculating the reduction of chlorophyll *a* (chl *a*) fluorescence (ex: 430 nm, em: 665 nm) of the diatom *S. marinoi* after 24 h incubation with *K. algicida* compared to a control. Reduction of chl *a* fluorescence was represented using the equation 1‐(chl *a*_T24_/chl *a*_T0_). *S. marinoi* was used as a target organism in the assays due to its susceptibility to *K. algicida* algicidal activity (Paul & Pohnert, 2011). To determine *K. algicida* algicidal activity, 100−200 µL of a culture was harvested via centrifugation for 10 min at 10,000 rpm and 13°C. Two sample volumes of filtered ASW medium were used to wash the pellets in duplicate, to remove any residual MB that could interfere with the bioassay. The washed *K. algicida* was then resuspended to a working OD_550_ of 0.08 in ASW. An equivalent volume of cell‐free MB (100–200 µL) was treated similarly, as a negative control for the algicidal activity assay, to account for potential media toxicity. one hundred and fifty microliter of exponentially growing *S. marinoi* was mixed with 50 µL of the resuspended *K. algicida* in a 96‐well microplate. The activity was determined by measuring the chl *a* fluorescence (Varioskan Flash, Thermo Fisher Scientific) of *S. marinoi* after 24 h.

For algicidal induction bioassays, inactive *K. algicida* was diluted (1:10 v/v) into filtered ASW and incubated overnight at 28°C and 80 rpm. The cultures were then analyzed for algicidal activity via the previously described method.

### 2.4 Test for metalloproteases

Algicidal bioassays were also undertaken in the presence of EDTA to exclude the involvement of metalloproteases. To this end, *K. algicida* was grown in 10 mL MB overnight at 28°C and shaking at 80 rpm. Cultures were diluted (1:20 v/v) in ASW and incubated overnight, at the same conditions. Cell‐free spent supernatant was collected for EDTA treatment by pelleting cultures through centrifugation for 10 min at 13,000 rpm and 13°C. EDTA‐2Na (Alfa Aesar) aqueous stock solution (100 mM) was added to a final concentration of 5 mM and an equivalent volume of H_2_O was added as a control. All samples were incubated in the dark for 15 min before the algicidal activity was assessed to allow time for the EDTA sequestration of metal ions.

For algicidal activity assays using recombinant proteins in ASW, cell‐free spent medium of exponentially growing *K. algicida*, diluted (1:20 v/v) in ASW then incubated for 24 h, was used as a positive control, and filtered ASW was used as a negative control. Fifty microliters of each treatment were added to 150 µL of *S. marinoi* cultures (*n* = 4) and chl *a* was measured at time 0 and 24 h to observe algicidal effects. To determine if algicidal activity is prevalent within the genus, *K. aestuariivivens*, *K. periserrulae*, and *Kordia* sp. were tested for algicidal properties, or inducibility thereof, as described above.

### 2.5 Fractionation of spent culture medium

To verify if other small molecules released by *K. algicida* contribute to the algicidal activity, we separated the spent *K. algicida* culture supernatant into a protein‐rich fraction (>3 kDa) and a small molecule‐rich fraction (<3 kDa). To this end, exponentially growing *K. algicida* in MB (~24 h) was inoculated into ASW (1:20 v/v) for overnight cultivation, at 28°C and shaking at 80 rpm. This allowed the accumulation of secreted algicidal compounds. The following day, cultures were centrifuged for 30 min at 9000 rpm and 15°C to produce a cell‐free spent medium. The protein‐rich fraction was separated from the small molecule‐rich fraction via centrifugal filtration for 30 min at 4500*g* and 4°C, using 3 kDa Amicon centrifugal tubes (Merk Millipore). The protein‐rich fraction was then washed with ASW medium and concentrated ×10 by centrifugation for 30 min at 4500*g* and 4°C. The flow‐through was applied to a 30 mg HLB SPE column, eluted with 3 mL MeOH (HPLC Grade), dried under N_2,_ and reconstituted to a ×10 concentration of the original volume with ASW. Algicidal activity assays were performed using the individual fractions, a recombination of the two fractions at a 1:1 ratio, as well as the unfiltered cell‐free spent medium as a positive control. For the assays, 50 µL of the treatments were added to 150 µL *S. marinoi* cultures, and chl *a* fluorescence was measured immediately after inoculation (0 h) and again 24 h later.

### 2.6 Preparation of bacterial samples for transcriptomic analysis

For transcriptomic analysis, *K. algicida* cultures were grown according to the section “*K. algicida* growth analysis” described above. To compare the active and inactive cultures of *K. algicida*, 3−6 mL aliquots of bacteria were collected at 30 and 75 h, based on results from the growth curve and activity analysis, as described above. Additionally, following the collection of 75 h samples, the inactive cultures were subjected to the induction of algicidal activity by dilution in ASW (1:10 v/v) followed by incubation at 28°C and shaking at 80 rpm for 24 h. Subsequently, an additional 3–6 mL sample was collected to represent induced activity. For each sample, cells were harvested via centrifugation for 10 min at 10,000 rpm and 4°C, flash frozen in liquid N_2_, and stored at −80°C until RNA extraction. For each sampling timepoint, bioassays were conducted concurrently to confirm algicidal activity. RNA extraction was carried out using an RNAeasy MiniKit (QIAGEN) following lysis via bead beating using a TissueLyser II (QIAGEN). Briefly, two cell pellet volumes of beads were added to each sample, and cells were broken for 3 min at 60 Hz and then cooled in liquid N_2_ (3−5 rounds). Lysed samples were then stored on ice and RNA extraction was carried out according to the manufacturer's recommendations. Extracted RNA was stored at −80°C and triplicate RNA samples of active and inactive *K. algicida* cultures were submitted to Novogene for comparative transcriptomic analysis. RNA aliquots from active, inactive, and induced cultures were kept for RT‐qPCR analysis.

### 2.7 RNA preparation, RNA‐seq library preparation, and sequencing

rRNA depletion was performed and total RNA was then ethanol precipitated. After fragmentation, the first strand of cDNA was synthesized using random hexamer primers. During the second strand cDNA synthesis, dUTPs were replaced with dTTPs in the reaction buffer. The directional library was ready after end repair, A‐tailing, adapter ligation, size selection, USER enzyme digestion, amplification, and purification. Each cDNA library was quantified using a Qubit Fluorometer and qPCR before pooling and sequencing. The pooled libraries were then sequenced as single‐end 100 bp reads on an Illumina HiSeq. 2000 system.

### 2.8 Transcriptomic data analysis

Raw data was processed through fastp (Chen et al., 2018) to trim Illumina adapters, poly‐N sequences, and low‐quality reads (<Q20). Reads were then quality filtered (base quality <5 across >50% of the read). Next, sample reads were aligned to the *K. algicida* OT‐1 reference genome, GenBank ABIB00000000.1, using Bowtie2 (Langmead & Salzberg, 2012) with mismatch parameter set to two, and other parameters set to default. Reads were then assembled according to the reference genome using Rockhopper (McClure et al., 2013). Novel gene transcripts were aligned to sequences in NCBI NR databases using Blastx (cutoff: *e* value <1 × 10^−5^). Gene expression levels were estimated via fragments per kilobase of transcript per million mapped reads (FPKM) using featureCounts (Liao et al., 2014).

### 2.9 RT‐qPCR

Aliquots of RNA samples sent for transcriptomic analysis were saved for in‐house analysis, with the RNA extracted from the additionally induced timepoint. RNA concentrations of active, inactive, and induced samples were measured using a Qubit reader with an RNA Broad Range assay kit (Thermo Fisher Scientific) and adjusted to the same starting concentration (18 ng/µL) using nuclease‐free water (Sigma‐Aldrich). cDNA was generated with a SuperScript IV VILO Master Mix with ezDNase enzyme digestion (Thermo Fisher Scientific) following the manufacturer's recommendations. Briefly, to remove contaminating gDNA, RNA samples were incubated at 37°C with ezDNase in ezDNase buffer for 2 min, then stored on ice. RNA samples were split for reverse transcription to cDNA. SuperScript IV VILO Master Mix was added to one sample and SuperScript IV VILO Master Mix No‐RT control was added to the other. Samples were then incubated at 25°C, for 10 min to anneal primers, at 50°C for 10 min to reverse transcribe RNA, and finally at 85°C for 5 min to inactivate the enzymes.

Target proteases were selected from the transcriptome analysis and primers were designed using Clone Manager 8, Professional Edition. For qPCR reactions, all primers were purchased from biomers.net GmbH. *KAOT1_RS10890* was amplified with forward primer ATCTATGCGCAAAGCTCGTG and reverse primer TGACTTCGGAGCTGACATTC. *KAOT1_RS09515* was amplified with forward primer AGGAATTGCGCCACATTCAG and reverse primer GTACGCTACACCGATAACAC. The *K. algicida* 16s rRNA gene was used as a housekeeping gene and amplified with forward primer GGTACTGTTGGATTGCATGATTC and reverse primer TCAGAGTTGCCTCCATTGTC. qPCR was performed by combining 0.5 µL of the cDNA template generated above with 0.5 µL of both forward and reverse primers, 5 µL SYBR Green Master Mix (Bio‐Rad Laboratories), and 3.5 µL H_2_O. Amplification was performed on a C1000 Touch Thermal Cycler CFX96 Real‐Time System (Bio‐Rad Laboratories) with the following program: 50°C for 2 min, 95°C for 2 min, followed by 40 cycles of 95°C for 15 s, 55°C for 15 s and 72°C for 1 min, then 60°C for 31 s followed by 60°C for 5 s ramped to 95°C for 0.5°C/cycle and 0.5°C/s for 70 cycles. Results were viewed using Bio‐Rad CFX Maestro 1.1.

### 2.10 Heterologous expression of AlpA1

For activity testing, AlpA1 was expressed in *E. coli*. First, genomic DNA was extracted from *K. algicida* using a DNeasy Blood & Tissue Kit (Qiagen). The *KAOT1_RS09515* gene, hereby known as *alpA1*, was amplified using the forward primer KaP1 (GAATTGGCCATAACGGACAGTATTACATCTTCTGATGAAG) and reverse primer KaP2 (GTAACTCGAGTTTTTTAGCATTTGGAGCTGTAAATCCG), designed to cover the complete sequence of the ORF except the first 60 bp. Amplification was achieved using Herculase II Fusion DNA Polymerase (Agilent Technologies) with 20 ng gDNA, 0.25 μM of each primer, 250 μM dNTPs and 4% (v/v) DMSO with the following program: 2 min initial denaturation at 95°C; 35 cycles of 20 s denaturation at 95°C, 20 s annealing at 55°C and 45 s elongation at 72°C; 3 min final elongation at 72°C. The PCR product was digested with 8000 U *Xho*I (New England Biolabs) and 10,000 U *Msc*I (New England Biolabs) in CutSmart buffer (New England Biolabs) for 1 h at 37°C. Vector pET‐26b (Novagen, Merck Millipore) was digested using the same restriction enzymes. The PCR product and open vector were purified innuPREP Gel Extraction Kit (Analytik Jena) and ligated using T4 Ligase (New England Biolabs) overnight at 4°C. The ligation product was transformed into electro‐competent *E. coli* Top10 (Invitrogen). The transformed cells were cultivated in LB medium for 1 h at 37°C and were then plated on LB agar plates with kanamycin for transformant selection. Plasmids were verified by restriction digest with *Xmn*I (New England Biolabs) and Sanger sequencing (Eurofins Genomics GmbH). The plasmid was then transformed into chemically competent *E. coli* Rosetta2(DE3) (Novagen, Merck). Transformants were verified by colony PCR with primers KaP1 and KaP2 and the above‐mentioned conditions for *alpA1* amplification. The resulting strain was called *E. coli* P390.

### 2.11 Expression and isolation of recombinant protein and BLASTp genera comparison

The expression and purification of AlpA1 were performed following a similar method as described in (Faezi et al., 2017). A single colony of *E. coli* P390 was grown overnight in a 10 mL LB medium supplemented with kanamycin (50 µg/mL) and then diluted into fresh 50 mL LB medium containing kanamycin to an OD_600_ of 0.05. When the culture achieved logarithmic growth phase ( ~ OD_600_ = 0.6), isopropyl β‐D‐1‐thiogalactopyranoside (IPTG) was added (final concentration 500 µM) and expression was induced for 4 h at 25°C and 180 rpm. A 30 mL aliquot was harvested at 4000*g* for 20 min at 4°C and re‐suspended in 2 mL PBS buffer (pH 7.4) for cell lysis. The resuspended cells were lysed via an ultrasonic probe with bursts of 10 s (100% power) followed by intervals of 30 s on ice. Cell debris was removed by centrifugation for 30 min at 9 000 rpm and 4°C. The supernatant was loaded onto a pre‐equilibrated HisPur Ni‐NTA spin column (Thermo Fisher Scientific) and purification under native conditions followed the manufacturer's instructions. Following elution, a buffer exchange with ASW was performed with 3 kDa MWCO Amicon centrifugal filters. The presence of the recombinant protein was confirmed via Western Blot. The recombinant protein was stored at −80°C for further bioassay. To search for AlpA1 homologs in the *Kordia* genus, the NCBI protein sequence of AlpA1 (WP_007094576.1) was used in a BLASTp query against all available Kordia genomes (taxID: 221065) (Altschul, 1997).

### 2.12 Statistical analysis

Algicidal activity between control and treatment cultures was compared with a paired, two‐tailed, Student's *t* test. The effect of EDTA on algicidal activity was determined by a one‐way analysis of variance (ANOVA). Post hoc comparisons were done with Tukey's honestly significant difference test. *p* < 0.05 were considered statistically significant. Statistical analysis was performed using GraphPad Prism (version 10.0.2). For transcriptomic data, differential expression analysis was performed using DESeq. 2 (Love et al., 2014). *p* values were adjusted using the Benjamini and Hochberg approach and genes with an adjusted *p* < 0.05 were assigned as differentially expressed. Functional annotation (GO terms and KEGG pathways) was performed using clusterProfiler (Yu et al., 2012).

## 3 RESULTS

### 3.1 Loss and restoration of algicidal activity of *K. algicida*

**Figure 1:**
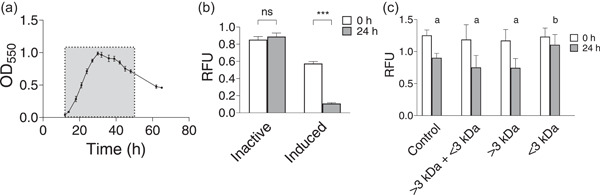
(a) Growth curve of *Kordia algicida* in MB. Gray shaded area denotes a period of algicidal activity (*n* = 4). (b) Inactive and induced activity of *K. algicida* was determined by change in chl a (RFU) of a diatom culture (*n* = 4). Samples were compared with a paired Student's *t* test with *p* values represented above the bars ( ***p < 0.001). Asterisks indicate significant differences between 0 and 24 h. (c) Activity of separated fractions of *K. algicida* spent medium: fraction enriched in small molecules (<3 kDa), fraction enriched in proteins (>3 kDa), and 1:1 recombination of both (>3 kDa + < 3 kDa), with whole spent medium used as algicidal control (*n* = 8). Activity was determined by the change in chl a (RFU) of a diatom culture. One‐way ANOVA was performed on 24‐h data, with significance letters indicated above the bars. All error bars indicate the standard deviation. MB, marine broth.

We performed a growth curve analysis with corresponding algicidal activity bioassays to understand the relationship between bacterial growth and algicidal activity. During the development of the culture, *K. algicida* becomes inactive, and algicidal activity can be rescued by exposure to nutrient starvation. Algicidal activity is present from the start of the culture until the death phase (gray‐shaded region in Figure 1a). To determine if the loss of activity was governed by quorum sensing effects, the inactive cultures at 72 h were inoculated into fresh MB. Despite the growth of the cultures, the algicidal activity was not recovered. We then rationalized that nutrient starvation may play a role in activating algicidal activity in the pressure for resource acquisition, and thus performed a subsequent incubation in nutrient‐limiting ASW medium (minimum ratio tested 1:10 v/v, MB:ASW). The data show that nutrient starvation could restore algicidal activity (Figure 1b).

A previous study by Paul and Pohnert (2011) reported that secreted proteases (>30 kDa) are, at least in part, responsible for the algicidal activity of *K. algicida*. To confirm the activity was due to secreted proteases, conditioned *K. algicida* medium was fractionated to obtain a fraction enriched in proteins (>3 kDa) and a fraction enriched in small molecules (<3 kDa). We, thus, generated protein‐enriched fractions and extracted and enriched small molecule fractions of the active spent medium of *K. algicida*. Application of these fractions in an algicidal activity assay showed that both the >3 kDa fraction, as well as the combined fractions, are algicidal, while the <3 kDa fraction is not (Figure 1c). Thus, confirming that the algicidal activity derives from the >3 kDa protein‐rich fraction, with little to no direct contribution by small molecules. Therefore, to determine which secreted enzymes could potentially be present during the algicidal phase, we proceeded with a transcriptomic analysis of active and inactive populations of *K. algicida*.

### 3.2 Transcriptomics reveals algicidal candidates

For the comparative transcriptomic analysis, samples were harvested at 30 h (point of maximum cell density and pronounced algicidal activity, hereafter “active”) and 75 h, well outside the algicidal window (hereafter “inactive”) (Figure 1a). An algicidal assay performed with an aliquot of each sample utilized for transcriptomic analysis confirmed an active and an inactive state were being compared (Appendix: Figure A2a). Between active and inactive states, 2589 genes displayed statistically significant differences in transcript levels (Appendix: Figure A2b). To search for secreted algicidal protease candidates, the list was narrowed down to genes that (1) were upregulated at the 30 h timepoint, (2) were annotated as protease or peptidase, and (3) contained a signal peptide. From this search, 24 protease/peptidase candidates were identified and the top 10 candidates, based on fold change analysis, are listed in Table 1.

**Table 1:** Top 10 differentially expressed candidate protease genes containing a signal peptide moiety.

Locus ID	NCBI accession	Predicted size (kDa)	Gene description	Fold change (active/inactive)	*p* Value
*KAOT1_RS10890*	WP_007094862.1	43	M57 family metalloprotease	13.396	3.25E‐23
*KAOT1_RS09515*	WP_007094576.1	45	S8 family serine peptidase, subtilisin‐like	12.507	4.08E‐35
*KAOT1_RS17795*	WP_009778466.1	37	Zinc metalloprotease	4.055	2.32E‐13
*KAOT1_RS14060*	WP_013869856.1	51	Insulinase family protein (Peptidase family M16)	3.593	2.27E‐07
*KAOT1_RS20965*	WP_013870093.1	55	S8/S53 family peptidase	3.406	3.96E‐05
*KAOT1_RS14065*	WP_013549898.1	77	Insulinase family protein (Peptidase family M16)	3.151	6.20E‐07
*KAOT1_RS16750*	WP_013750727.1	47	Peptidoglycan DD‐metalloendopeptidase (Peptidase family M23)	3.106	3.79E‐08
*KAOT1_RS14920*	WP_013869438.1	49	Peptidoglycan DD‐metalloendopeptidase (Peptidase family M23)	3.090	1.80E‐05
*KAOT1_RS16770*	WP_162014347.1	68	SprT family zinc‐dependent metalloprotease	3.070	8.20E‐05
*KAOT1_RS17420*	WP_007096197.1	23	Cysteine peptidase family C39 domain‐containing protein	2.861	3.82E‐06

**Figure 2:**
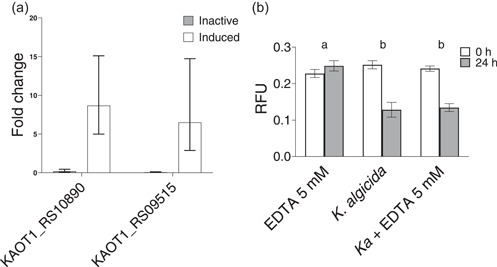
(a) qPCR of algicidal protease gene candidates in active, inactive, and induced states (*n* = 3). Error bars represent the confidence interval of the fold change. (b) Algicidal activity of *Kordia algicida* spent medium treated with EDTA (*Ka* + EDTA 5 mM), EDTA 5 mM in ASW (negative control), and spent medium of *K. algicida* (positive control) (*n* = 4), measured by change in chl *a* (RFU). One‐way ANOVA was performed on data between time measurements among different treatments with significance letters indicated above the bars. Error bars indicate the standard deviation. ASW, artificial seawater media.

**Figure A1:**
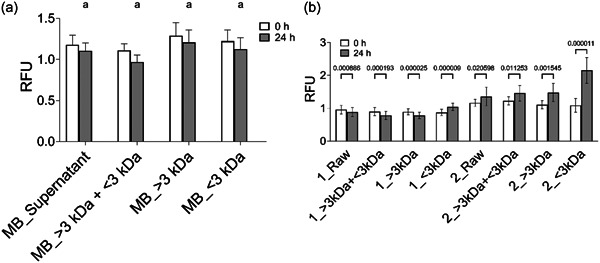
Controls and stability assays for algicidal activity measured in Figure 1c. (a) Medium blank controls for fractionation of spent medium. The blank medium was fractioned and evaluated for algicidal activity of the <3 kDa fraction, the >3 kDa fraction, and the 1:1 (v/v) recombination of both ( > 3 kDa + <3 kDa), with the unfractionated medium (MB_Supernatant) used as algicidal control (*n* = 8). One‐way ANOVA was performed on 24‐h data, with significance letters indicated above the bars. (b) Stability of algicidal fractions (from Figure 1c) at room temperature measured over 2 days, represented by 1_ or 2_. Samples were compared with a paired Student's *t* test with *p* values represented above the bars. All error bars indicate the standard deviation (*n* = 8).

**Figure A2:**
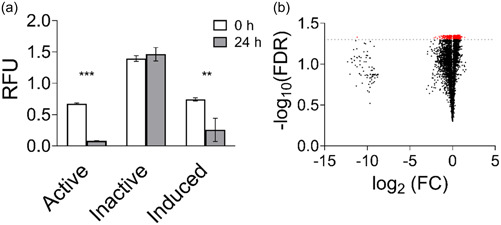
(a) Activity of *Kordia algicida* cells before RNA extraction, active (30 h), inactive (75 h), and induced (*n* = 4). Samples were compared with a paired Student's *t* test with *p* values represented above the bars (**p < 0.01, ***p < 0.001). Error bars indicate the standard deviation. (b) Volcano plot showing fold change of transcripts present in the transcriptomic analysis. Comparison was done between active (30 h) and inactive (75 h) samples (*n* = 3).

**Figure A3:**
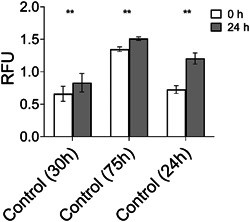
Algicidal activity of medium controls for samples submitted for transcriptomic analysis (*n* = 4). Samples were compared with a paired Student's *t* test with *p* values represented above the bars (**p < 0.01). Error bars indicate the standard deviation.

**Figure A4:**
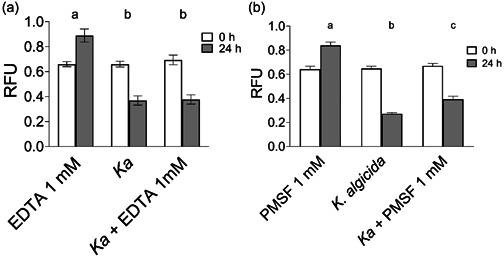
(a) Algicidal activity assays in the presence of 1 mM EDTA (*n* = 4). (b) Algicidal activity assays in the presence of 1 mM PMSF (*n* = 4). One‐way ANOVA was performed to compare the samples with the significance letters indicated above the bars. Error bars indicate the standard deviation.

To confirm the transcriptomic results, and further narrow down the list of candidates, the transcript levels of the two largest fold change protease/peptidases were measured by RT‐qPCR. For this experiment, we used saved aliquots of RNA from the samples submitted for transcriptomic analysis of active and inactive *K. algicida*. Additionally, we analyzed RNA samples from induced *K. algicida*. These were generated by diluting the inactive (75 h) *K. algicida* in ASW medium for 24 h, thus inducing algicidal activity (Appendix: Figure A2a). We hypothesized that candidates for algicidal proteases would show a pattern of higher expression in the active phase (30 h), lower expression in the inactive phase (75 h), and an increase in the induced sample. This pattern was observed for both *KAOT1_RS10890* and *KAOT1_RS09515* (Figure 2a). (Appendix: Figures A1, A3, A4).

### 3.3 *KAOT1_RS09515* is an algicidal protease of *K. algicida*

The transcripts of *KAOT1_RS10890* showed the highest fold change between active and inactive phases in the transcriptomic experiment and between inactive and induced phases in the RT‐qPCR experiment. *KAOT1_RS10890* encodes an M57 family metalloprotease. Therefore, we hypothesized that if this metalloprotease is responsible for the observed algicidal activity, the addition of EDTA should show an inhibitory effect through the broad inactivation of metalloproteases. However, this was not the case. Compared to the control, the addition of 5 mM EDTA does not significantly change the algicidal activity of the *K. algicida* cell‐free supernatant (Figure 2b).

**Figure 3:**
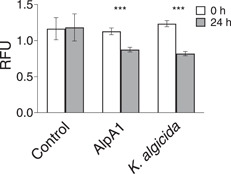
Algicidal activity, measured by the change in chl *a* (RFU) of a diatom culture. Cultures were either exposed to ASW medium (negative control), the recombinant protein AlpA1, or the spent medium of *Kordia algicida* in ASW. Error bars indicate the standard deviation (*n* = 4). Samples were compared with a paired Student's *t* test with *p* values represented above the bars (*** *p* < 0.001). Asterisks indicate significant differences between control and treatment at 24 h. ASW, artificial seawater media.

**Figure A5:**
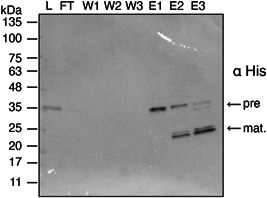
Anti‐6xHis‐tag immunoblot of the purification of recombinant AlpA1. Pre‐ and mature protein bands are indicated with arrows. E, elution; FT, flow through; L, lysate; W, wash.

**Figure A6:**
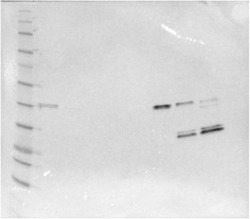
Raw picture of Figure A5.

We, thus, focused our efforts on the S8 family serine peptidase *KAOT1_RS09515*. To test whether *KAOT1_RS09515* had algicidal activity, the gene was heterologously expressed in *E. coli* with a 6xHis tag for purification. The presence of a protein with approximately 45 kDa was observed in the elution lanes (Appendix: Figures A5 and A6), which was consistent with the full length of KAOT1_RS09515. Additionally, a second prominent band was present at approximately 24 kDa, which likely indicates posttranslational activation of the recombinant serine peptidase. An aliquot of the elution fraction was buffer exchanged to ASW and applied in an algicidal assay. The fraction containing KAOT1_RS09515 exhibited algicidal activity similar to the *K. algicida* cell‐free supernatant (Figure 3). Therefore, we named *KAOT1_RS09515*, the algicidal protease encoding gene, *alpA1* (algicidal protease 1).

### 3.4 Algicidal activity is unique to *K. algicida* OT‐1

**Figure 4:**
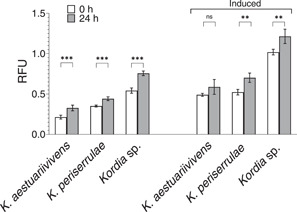
Algicidal activity of three *Kordia* species, measured by the change in chl *a* (RFU) in diatom cultures (*n* = 4). Algicidal activity with and without induction was determined in conditions identical to *Kordia algicida*. Samples were compared with a paired Student's *t* test with *p* values represented above the bars (**p < 0.01, ***p < 0.001). Asterisks indicate significant differences between 0 and 24 h. Error bars indicate the standard deviation.

*K. aestuariivivens, K. periserrulae*, and *Kordia sp*. were tested for algicidal activity and algicidal inducibility in the same manner as *K. algicida*. For all three strains, the initial evaluation of exponentially growing cultures in MB showed no reduction of diatom chl *a* fluorescence (Figure 4) and remained inactive following induction in ASW (Figure 4).

**Figure A7:**
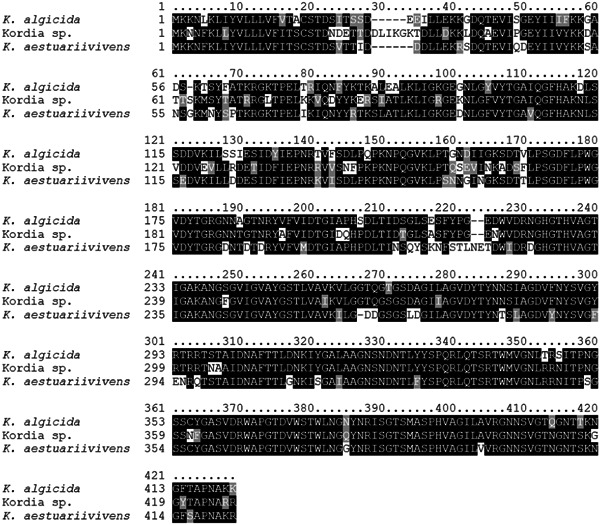
Alignment of AlpA1 (*Kordia algicida)* with homologues from *Kordia* sp. and *Kordia aestuariivivens*.

We hypothesized that the unique algicidal ability of *K. algicida* compared to other members of the genus is likely linked to specialized genes evolved in this species, which may be visible in comparisons with nonalgicidal species. BLASTp identified two homologs of AlpA1 with 78% similarity in *Kordia* sp. and *K. aestuariivivens* (Table 2). However, these two species showed no algicidal activity in our bioassays. An alignment of the three proteins shows that there are several instances where amino acids are unique to AlpA1 that could account for the difference in activity (Figure A7). Interestingly, in both AlpA1 homologs, a potentially conserved serine at position 209 is replaced by a proline.

**Table 2:** BLASTp search of AlpA1 homology in the *Kordia* genus.

Scientific name	NCBI accession	Percent identity (%)	*e* Value
*Kordia algicida* OT‐1	WP_007094576.1	100.00	0.0
*Kordia sp*. ALOHA_ZT_18	MCH2196544.1	78.69	0.0
*Kordia aestuariivivens* YSTF‐M3	WP_187560797.1	78.30	0.0
*Kordia aestuariivivens* YSTF‐M3	WP_187560671.1	34.31	4.00E‐45
*Kordia aestuariivivens* YSTF‐M3	WP_187562711.1	30.51	3.00E‐37
*Kordia* sp. ALOHA_ZT_18	MCH2196921.1	35.42	5.00E‐14

## 4 DISCUSSION

The observation of an algicidal active and inactive phase during the growth of *K. algicida* enabled a transcriptomic approach to identify potential genes encoding proteases. Evaluation of differentially expressed genes encoding secreted proteins, validation with RT‐qPCR, and inhibition assays led to the discovery of the protease gene *alpA1* (*KAOT1_RS09515*). The recombinant protein, AlpA1, generated from transformation and expression in *E. coli*, showed in vivo algicidal activity. Our findings were further supported by the fact that, in the *Kordia* genus, this gene is unique to *K. algicida*.

Algicidal activity in *K. algicida* is a highly regulated process. A previous study has suggested that algicidal activity in *K. algicida* is regulated by a quorum‐sensing mechanism. Based on our results, we cannot exclude this is the case. However, algicidal activity is also regulated independently of quorum‐sensing, for example by environmental factors (e.g., nutrient limitation) (Paul & Pohnert, 2011). Our studies show an additional dependence of algicidal activity on nutrient availability. A nutrient‐dependent activity has been previously reported for laboratory‐grown strains of algicidal bacteria, favoring replete conditions for the excretion of algicides (Mayali & Doucette, 2002; Roth et al., 2008). When we grew bacteria in nutrient‐rich medium, activity declined following stationary phase but could be restored by exchanging the spent culture medium with ASW medium that contains no organic substrate. Thus, activity is not only found under nutrient repletion, but also in nutrient‐poor ASW medium. This indicates a complex nutrient‐ and quorum‐sensing‐dependent regulation. This pattern might reflect the situation in the oceans where, in nutrient‐poor conditions, bacteria utilize algicidal proteases to lyse an algal cell, thus creating a nutrient‐rich local environment where there is no need for further protease production. Once these resources are depleted, or the bacterium is separated from the nutrient hotspot, it re‐initiates algicidal production to target the next cell. This induction of protease release in the open, nutrient‐limited, water likely represents a strategy to save metabolic investment in algicide production. Overall, this demonstrates that the regulatory mechanisms of algicidal activity are complex, and further work is needed to untangle them.

AlpA1 is part of the S8 family serine peptidases, which have potential multifunctionality as algicides and facilitators of uptake by extracellular degradation of biopolymers, such as proteins (Meyer et al., 2017). The involvement of a serine protease in algicidal activity has only been documented in the algicidal effects of *Pseudoalteromonas* sp. (Lee et al., 2000, 2002). In this species, a metalloprotease also showed algicidal activity, albeit with six‐fold less activity than the serine protease (Kohno et al., 2007; Lee et al., 2000, 2002). Given the exceptionally high fold‐change of AlpA1 during the active phase of the bacteria, we consider it a major player in the algicidal activity. Further evaluation is needed to confirm the extent of the contribution from other candidate enzymes. These other proteases may of course be indirectly involved in the exploitation of resources. This is not an uncommon mode of action, as it is becoming more apparent that algicidal activity can be associated with multiple components working together to make the predatory lifestyle effective. In this case, it likely involves nutrient acquisition from targeted algae (Coyne et al., 2022; Jeong & Son, 2021; Rose et al., 2021; Wang & Seyedsayamdost, 2017; Zhang et al., 2018). It is reasonable to conclude that the full algicidal mode of *K. algicida* may involve a repertoire of other enzymes, which act together with AlpA1. Based on our size fractionation experiments (Figure 1c), and results by Paul and Pohnert (2013), low molecular weight specialized metabolites will likely not support the algicidal activity but might influence competition or cooperation with other bacteria.

Recent studies have favored the application of molecular techniques to describe algicidal bacterial‐algal interactions, but offer descriptive characteristics of the interactions, rather than the identification of direct factors involved in the attack (Hu et al., 2021; Zhang et al., 2021, 2023). In addition, the existing transcriptomic studies were dependent on the exposure of algicidal bacteria to hosts, which introduces an additional layer of complexity to data analysis (Zhang et al., 2022b; Zhang et al., 2023). These studies identified major pathways involved in the algicidal process such as energy production and amino acid metabolism but did not present specific gene candidates for algicidal activity (Zhang et al., 2022b; Zhang et al., 2023). In the case of *K. algicida*, no host‐dependent algicide production was observed, but rather a growth state dependency of activity (Paul & Pohnert, 2011). In this study, understanding the regulation of algicidal activity in *Kordia* was central to the transcriptomic analysis. This allowed us to analyze the transcriptional regulation without the added complexity of induced interactions from co‐culturing experiments between bacteria and host algae. The candidate pool could thereby be narrowed down, reducing competing responses from the diverse chemical signaling in response to host algae and the associated microbiome.

Many lytic bacteria are species or genus‐specific, but no connection has been found between algicidal activity and phylogeny (Doucette et al., 1999). The *Kordia* genus belongs to the *Flavobacteriaceae* family and has only been discovered and explored in the last two decades. Though algicidal bacteria of the *Flavobacteriaceae* family mostly target diatoms and some dinoflagellates (Coyne et al., 2022), *K. algicida* exhibits an exceptionally broad host range including the raphidophyte *Heterosigma akashiwo* (Sohn et al., 2004) and the coccolithophore *Emiliania huxleyi* as observed in our lab (data not published). To explore the algicidal potential of *Kordia* spp., we sought to expand the investigation to other *Kordia* species, including *K. periserrulae*, the closest genetic relative of *K. algicida* (Lee et al., 2011). Remarkably, none of the strains tested were algicidal in our bioassay. BLASTp comparisons of AlpA1 with other proteases within the *Kordia* genera showed close homology to proteins in *Kordia* sp. and *K. aestuariivivens*, with a difference of less than 30%. In both these cases, the serine at position 209, belonging to the catalytic Ser/His/Asp triad, is replaced by proline in both AlpA1 homologs, which could explain the difference in activity amongst the homologs (Ekici et al., 2008). *K. algicida* has thus adopted a unique lifestyle among the members of the *Kordia* genus. These results also reiterate the lack of correlation between phylogeny and algicidal properties observed by Wang et al. (2020).

Despite much research effort, the ecological role of algicidal bacteria in controlling algal blooms lacks concrete evidence, mainly caused by the lack of appropriate tools for observation (Skerratt et al., 2002). Transcriptomic analysis allows tracking bacterial activity, but without definitive algicidal transcripts to monitor, there is still ambiguity in the data interpretation. By elucidating a specific algicidal transcript from *K. algicida*, we introduce the possibility of real‐time monitoring of algicidal protease gene expression in algal blooms, as in Masan Bay where it was discovered. The newly identified protease, and the transgenic lines here generated, now also provide an opportunity to clarify the ecological role of algicidal bacteria. This will answer the longstanding question of whether algicidal bacteria play an active role in bloom regulation or are merely opportunistic bystanders waiting for the right conditions to flourish.

## AUTHOR CONTRIBUTIONS

**Kristy S. Syhapanha**: conceptualization (lead); writing—original draft (lead); formal analysis (lead); writing—review and editing (equal); **David A. Russo**: conceptualization (supporting); writing—original draft (supporting); formal analysis (supporting); writing—review and editing (equal); **Yun Deng**: conceptualization (supporting); formal analysis (supporting); writing—review and editing (equal); **Nils Meyer**: formal analysis (supporting); **Remington X. Poulin**: conceptualization (supporting); **Georg Pohnert**: conceptualization (lead); review and editing (equal).

## CONFLICT OF INTEREST STATEMENT

None declared.

## ETHICS STATEMENT

None required.

## Data Availability

The transcriptomics data set generated and analyzed during the current study is available in the Zenodo repository at https://doi.org/10.5281/zenodo.8276193.

## APPENDIX A

Table A1

**Table A1:** Measurement of relative gene expression by qPCR.

Fold change analysis ($2^{-\Delta \Delta C_{q}}$)			
	Active–active	Inactive–active	Induced–inactive
KAOT1_RS09515	1	0.0675	6.5055
KAOT1_RS10890	1	0.1957	8.6838

## References

[mbo31387-bib-0001] Altschul, S. (1997). Gapped BLAST and PSI‐BLAST: A new generation of protein database search programs. Nucleic Acids Research, 25, 3389–3402. 9254694 10.1093/nar/25.17.3389 PMC146917

[mbo31387-bib-0002] Azam, F. , Fenchel, T. , Field, J. , Gray, J. , Meyer‐Reil, L. , & Thingstad, F. (1983). The ecological role of water‐column microbes in the sea. Marine Ecology Progress Series, 10, 257–263.

[mbo31387-bib-0003] Banin, E. , Khare, S. K. , Naider, F. , & Rosenberg, E. (2001). Proline‐rich peptide from the coral pathogen *Vibrio shiloi* that inhibits photosynthesis of zooxanthellae. Applied and Environmental Microbiology, 67, 1536–1541. 11282602 10.1128/AEM.67.4.1536-1541.2001 PMC92766

[mbo31387-bib-0004] Bigalke, A. , Meyer, N. , Papanikolopoulou, L. A. , Wiltshire, K. H. , & Pohnert, G. (2019). The algicidal bacterium *Kordia algicida* shapes a natural plankton community. Applied and Environmental Microbiology, 85, e02779. 30737345 10.1128/AEM.02779-18 PMC6585488

[mbo31387-bib-0005] Chen, S. , Zhou, Y. , Chen, Y. , & Gu, J. (2018). fastp: An ultra‐fast all‐in‐one FASTQ preprocessor. Bioinformatics, 34, i884–i890. 30423086 10.1093/bioinformatics/bty560 PMC6129281

[mbo31387-bib-0006] Chen, W. , Lin, C. , & Sheu, S. (2010). Investigating antimicrobial activity in *Rheinheimera* sp. due to hydrogen peroxide generated by l‐lysine oxidase activity. Enzyme and Microbial Technology, 46, 487–493. 25919624 10.1016/j.enzmictec.2010.01.006

[mbo31387-bib-0007] Chen, W. , Sheu, F. , & Sheu, S. (2011). Novel L‐amino acid oxidase with algicidal activity against toxic cyanobacterium *Microcystis aeruginosa* synthesized by a bacterium *Aquimarina* sp. Enzyme and Microbial Technology, 49, 372–379. 22112563 10.1016/j.enzmictec.2011.06.016

[mbo31387-bib-0008] Coyne, K. J. , Wang, Y. , & Johnson, G. (2022). Algicidal bacteria: A review of current knowledge and applications to control harmful algal blooms. Frontiers in Microbiology, 13, 13. 10.3389/fmicb.2022.871177 PMC9022068 35464927

[mbo31387-bib-0009] Doucette, G. J. , Mcgovern, E. R. , & Babinchak, J. A. (1999). Algicidal bacteria active against *Gymnodinium breve* (Dinophyceae). I. Bacterial isolation and characterization of killing activity^1,3^ . Journal of Phycology, 35, 1447–1454.

[mbo31387-bib-0010] Dow, L. (2021). How do quorum‐sensing signals mediate algae–bacteria interactions? Microorganisms, 9, 1391. 34199114 10.3390/microorganisms9071391 PMC8307130

[mbo31387-bib-0011] Ekici, Ö. D. , Paetzel, M. , & Dalbey, R. E. (2008). Unconventional serine proteases: Variations on the catalytic Ser/His/Asp triad configuration. Protein Science, 17, 2023–2037. 18824507 10.1110/ps.035436.108 PMC2590910

[mbo31387-bib-0012] Faezi, S. , Bahrmand, A. R. , Mahdavi, M. , Siadat, S. D. , Nikokar, I. , & Sardari, S. (2017). Development of a novel anti‐adhesive vaccine against *Pseudomonasaeruginosa* targeting the C‐terminal disulfide loop of the pilin protein. International Journal of Molecular and Cellular Medicine, 6, 96–108. 28890886 10.22088/acadpub.BUMS.6.2.4 PMC5581551

[mbo31387-bib-0013] Hibayashi, R. , & Imamura, N. (2003). Action mechanism of a selective anti‐cyanobacterial compound, argimicin A. The Journal of Antibiotics, 56, 154–159. 12715875 10.7164/antibiotics.56.154

[mbo31387-bib-0014] Hu, T. , Wang, S. , Shan, Y. , Zhang, Y. , Zhu, Y. , & Zheng, L. (2021). Complete genome of marine microalgae associated algicidal bacterium *Sulfitobacter pseudonitzschiae* H46 with quorum sensing system. Current Microbiology, 78, 3741–3750. 34459935 10.1007/s00284-021-02632-4

[mbo31387-bib-0015] Imai, I. , Sunahara, T. , Nishikawa, T. , Hori, Y. , Kondo, R. , & Hiroishi, S. (2001). Fluctuations of the red tide flagellates *Chattonella* spp. (Raphidophyceae) and the algicidal bacterium *Cytophaga* sp. in the Seto Inland Sea, Japan. Marine Biology, 138, 1043–1049.

[mbo31387-bib-0016] Imamura, N. , Motoike, I. , Noda, M. , Adachi, K. , Konno, A. , & Fukami, H. (2000). Argimicin A, a novel anti‐cyanobacterial compound produced by an algae‐lysing bacterium. The Journal of Antibiotics, 53, 1317–1319. 11213296 10.7164/antibiotics.53.1317

[mbo31387-bib-0050] Jeong, S.‐Y. , & Son, H.‐J. (2021). Effects of mycosubtilin homolog algicides from a marine bacterium, Bacillus sp. SY‐1, against the harmful algal bloom species Cochlodinium polykrikoides. Journal of Microbiology, 59, 389–400. 33779952 10.1007/s12275-021-1086-8

[mbo31387-bib-0017] Kim, J.‐D. , Kim, J.‐Y. , Park, J.‐K. , & Lee, C.‐G. (2009). Selective control of the *Prorocentrum minimum* harmful algal blooms by a novel algal‐lytic bacterium *Pseudoalteromonas haloplanktis* AFMB‐008041. Marine Biotechnology, 11, 463–472. 19048341 10.1007/s10126-008-9167-9

[mbo31387-bib-0018] Kim, M. , Yoshinaga, I. , Imai, I. , Nagasaki, K. , Itakura, S. , Uchida, A. , & Ishida, Y. (1998). A close relationship between algicidal bacteria and termination of *Heterosigma akashiwo* (Raphidophyceae) blooms in Hiroshima Bay, Japan. Marine Ecology Progress Series, 170, 25–32.

[mbo31387-bib-0019] Kohno, D. , Sakiyama, Y. , Lee, S.‐O. , Takiguchi, N. , Mitsutani, A. , Kitaguchi, H. , & Kato, J. (2007). Cloning and characterization of a gene encoding algicidal serine protease from *Pseudoalteromonas* sp. strain. Journal of Environmental Biotechnology, 7, A2899.

[mbo31387-bib-0020] Langmead, B. , & Salzberg, S. L. (2012). Fast gapped‐read alignment with Bowtie 2. Nature Methods, 9, 357–359. 22388286 10.1038/nmeth.1923 PMC3322381

[mbo31387-bib-0021] Lee, H. S. , Kang, S. G. , Kwon, K. K. , Lee, J.‐H. , & Kim, S.‐J. (2011). Genome sequence of the algicidal bacterium *Kordia algicida* OT‐1. Journal of Bacteriology, 193, 4031–4032. 21622754 10.1128/JB.05241-11 PMC3147531

[mbo31387-bib-0022] Lee, S. , Kato, J. , Takiguchi, N. , Kuroda, A. , Ikeda, T. , Mitsutani, A. , & Ohtake, H. (2000). Involvement of an extracellular protease in algicidal activity of the marine bacterium *Pseudoalteromonas* sp. strain A28. Applied and Environmental Microbiology, 66, 4334–4339. 11010878 10.1128/aem.66.10.4334-4339.2000 PMC92304

[mbo31387-bib-0023] Lee, S.‐O. , Kato, J. , Nakashima, K. , Kuroda, A. , Ikeda, T. , Takiguchi, N. , & Ohtake, H. (2002). Cloning and characterization of extracellular metal protease gene of the algicidal marine bacterium *Pseudoalteromonas* sp. strain A28. Bioscience, Biotechnology, and Biochemistry, 66, 1366–1369. 12162559 10.1271/bbb.66.1366

[mbo31387-bib-0024] Li, Y. , Lei, X. , Zhu, H. , Zhang, H. , Guan, C. , Chen, Z. , Zheng, W. , Fu, L. , & Zheng, T. (2016). Chitinase producing bacteria with direct algicidal activity on marine diatoms. Scientific Reports, 6, 21984. 26902175 10.1038/srep21984 PMC4763246

[mbo31387-bib-0025] Liao, Y. , Smyth, G. K. , & Shi, W. (2014). featureCounts: An efficient general purpose program for assigning sequence reads to genomic features. Bioinformatics, 30, 923–930. 24227677 10.1093/bioinformatics/btt656

[mbo31387-bib-0026] Love, M. , Huber, W. , & Anders, S. (2014). Moderated estimation of fold change and dispersion for RNA‐seq data with DESeq. 2. Genome Biology, 15, 550. 25516281 10.1186/s13059-014-0550-8 PMC4302049

[mbo31387-bib-0027] Maier, I. , & Calenberg, M. (1994). Effect of extracellular Ca2+ and Ca2+‐antagonists on the movement and chemoorientation of male gametes of *Ectocarpus siliculosus* (Phaeophyceae). Botanica Acta, 107, 451–460.

[mbo31387-bib-0028] Mayali, X. , & Azam, F. (2004). Algicidal bacteria in the sea and their impact on algal Blooms. Journal of Eukaryotic Microbiology, 51, 139–144. 15134248 10.1111/j.1550-7408.2004.tb00538.x

[mbo31387-bib-0029] Mayali, X. , & Doucette, G. J. (2002). Microbial community interactions and population dynamics of an algicidal bacterium active against *Karenia brevis* (Dinophyceae). Harmful algae, 1, 277–293.

[mbo31387-bib-0030] Mayali, X. , Franks, P. J. S. , Tanaka, Y. , & Azam, F. (2008). Bacteria‐induced motility reduction in lingulodinium polyedrum (dinophyceae)1. Journal of Phycology, 44, 923–928. 27041610 10.1111/j.1529-8817.2008.00549.x

[mbo31387-bib-0031] McClure, R. , Balasubramanian, D. , Sun, Y. , Bobrovskyy, M. , Sumby, P. , Genco, C. A. , Vanderpool, C. K. , & Tjaden, B. (2013). Computational analysis of bacterial RNA‐Seq data. Nucleic Acids Research, 41, e140. 23716638 10.1093/nar/gkt444 PMC3737546

[mbo31387-bib-0032] Meyer, N. , Bigalke, A. , Kaulfuß, A. , & Pohnert, G. (2017). Strategies and ecological roles of algicidal bacteria. FEMS Microbiology Reviews, 41, 880–899. 28961821 10.1093/femsre/fux029

[mbo31387-bib-0033] Onishi, Y. , Tuji, A. , Yamaguchi, A. , & Imai, I. (2021). Distribution of growth‐inhibiting bacteria against the toxic dinoflagellate *Alexandrium catenella* (Group I) in Akkeshi‐Ko Estuary and Akkeshi Bay, Hokkaido, Japan. Applied Sciences, 11, 172.

[mbo31387-bib-0034] Paul, C. , & Pohnert, G. (2011). Interactions of the algicidal bacterium *Kordia algicida* with diatoms: Regulated protease excretion for specific algal lysis. PLoS ONE, 6, e21032. 21695044 10.1371/journal.pone.0021032 PMC3117869

[mbo31387-bib-0035] Paul, C. , & Pohnert, G. (2013). Induction of protease release of the resistant diatom *Chaetoceros didymus* in response to lytic enzymes from an algicidal bacterium. PLoS ONE, 8, e57577. 23469204 10.1371/journal.pone.0057577 PMC3587623

[mbo31387-bib-0051] Rose, M. M. , Scheer, D. , Hou, Y. , Hotter, V. S. , Komor, A. J. , Aiyar, P. , Scherlack, K. , Vergara, F. , Yan, Q. , & Loper, J. E. (2021). The bacterium Pseudomonas protegens antagonizes the microalga Chlamydomonas reinhardtii using a blend of toxins. Environmental Microbiology, 23, 5525–5540. 34347373 10.1111/1462-2920.15700

[mbo31387-bib-0036] Roth, P. , Twiner, M. , Mikulski, C. , Barnhorst, A. , & Doucette, G. (2008). Comparative analysis of two algicidal bacteria active against the red tide dinoflagellate *Karenia brevis* . Harmful Algae, 7, 682–691.

[mbo31387-bib-0037] Sakata, T. , Yoshikawa, T. , & Nishitarumizu, S. (2011). Algicidal activity and identification of an algicidal substance produced by marine *Pseudomonas* sp. C55a‐2. Fisheries Science, 77, 397–402.

[mbo31387-bib-0038] Skerratt, J. , Bowman, J. , Hallegraeff, G. , James, S. , & Nichols, P. (2002). Algicidal bacteria associated with blooms of a toxic dinoflagellate in a temperate Australian estuary. Marine Ecology Progress Series, 244, 1–15.

[mbo31387-bib-0039] Sohn, J. , Lee, J.‐H. , Yi, H. , Chun, J. , Bae, K. , Ahn, T.‐Y. , & Kim, S.‐J. (2004). *Kordia algicida* gen. nov., sp. nov., an algicidal bacterium isolated from red tide. International Journal of Systematic and Evolutionary Microbiology, 54, 675–680. 15143006 10.1099/ijs.0.02689-0

[mbo31387-bib-0040] Ternon, E. , Wang, Y. , & Coyne, K. (2018). Small polar molecules: A challenge in marine chemical ecology. Molecules, 24, 135. 30602708 10.3390/molecules24010135 PMC6337545

[mbo31387-bib-0041] Wang, M. , Chen, S. , Zhou, W. , Yuan, W. , & Wang, D. (2020). Algal cell lysis by bacteria: A review and comparison to conventional methods. Algal Research, 46, 101794.

[mbo31387-bib-0052] Wang, R. , & Seyedsayamdost, M. R. (2017). Roseochelin B, an algaecidal natural product synthesized by the roseobacter Phaeobacter inhibens in response to algal sinapic acid. Organic Letters, 19, 5138–5141. 28920692 10.1021/acs.orglett.7b02424

[mbo31387-bib-0042] Wu, Y. , Liu, J. , Yang, L. , Chen, H. , Zhang, S. , Zhao, H. , & Zhang, N. (2011). Allelopathic control of cyanobacterial blooms by periphyton biofilms. Environmental Microbiology, 13, 604–615. 21054736 10.1111/j.1462-2920.2010.02363.x

[mbo31387-bib-0043] Yu, G. , Wang, L.‐G. , Han, Y. , & He, Q.‐Y. (2012). clusterProfiler: An R package for comparing biological themes among gene clusters. OMICS: A Journal of Integrative Biology, 16, 284–287. 22455463 10.1089/omi.2011.0118 PMC3339379

[mbo31387-bib-0053] Zhang, B. , Hu, S. , Sun, S. , Fang, T. , Yu, Y. , Sun, X. , & Xu, N. (2022b). Transcriptomic analysis provides insights into the algicidal mechanism of cocamidopropyl betaine against the red tide microalgae Skeletonema costatum. Marine Environmental Research, 183, 105838. 36525828 10.1016/j.marenvres.2022.105838

[mbo31387-bib-0044] Zhang, B. , Yang, Y. , He, W. , & Liu, W. (2023). Algicidal process and mechanisms of *Enterobacter hormaechei* F2 revealed by an integrated transcriptomic and metabolomic approach. Genomics, 115, 110586. 36796656 10.1016/j.ygeno.2023.110586

[mbo31387-bib-0054] Zhang, F. , Ye, Q. , Chen, Q. , Yang, K. , Zhang, D. , Chen, Z. , Lu, S. , Shao, X. , Fan, Y. , Yao, L. , Ke, L. , Zheng, T. , & Xu, H. (2018). Algicidal Activity of Novel Marine Bacterium Paracoccus sp. Strain Y42 against a Harmful Algal‐Bloom‐Causing Dinoflagellate, Prorocentrum donghaiense. Applied and Environmental Microbiology, 84, e01015–e01018. 30054369 10.1128/AEM.01015-18 PMC6147001

[mbo31387-bib-0045] Zhang, Y. , Chen, D. , Cai, J. , Zhang, N. , Li, F. , Li, C. , & Huang, X. (2021). Complete genome sequence analysis of *Brevibacillus laterosporus* Bl‐zj reflects its potential algicidal response. Current Microbiology, 78, 1409–1417. 33649996 10.1007/s00284-021-02378-z

[mbo31387-bib-0046] Zhang, Y. , Li, J. , Hu, Z. , Chen, D. , Li, F. , Huang, X. , & Li, C. (2022). Transcriptome analysis reveals the algicidal mechanism of *Brevibacillus laterosporus* against *Microcystis aeruginosa* through multiple metabolic pathways. Toxins, 14, 492. 35878230 10.3390/toxins14070492 PMC9320710

[mbo31387-bib-0047] Zhou, Q. , Zhang, Y. , Han, S. , Wang, Y. , Qin, H. , & Zhang, Z. (2021). Physiological responses of *Microcystis aeruginosa* to extracellular degradative enzymes and algicidal substance from heterotrophic bacteria. Polish Journal of Environmental Studies, 30, 2947–2955.

